# Operationalizing inclusion through drama: the effects of inclusive children's literature on empathic tendencies

**DOI:** 10.3389/fpsyg.2026.1836003

**Published:** 2026-06-01

**Authors:** Ülkü Çoban Sural, Zehra Yaşar Sağlık

**Affiliations:** 1 Faculty of Education, Department of Primary Education, Karamanoglu Mehmetbey University, Karaman, Türkiye; 2 Faculty of Education, Department of Primary Education, Hasan Kalyoncu University, Gaziantep, Türkiye

**Keywords:** creative drama, empathy, inclusive children's literature, primary education, prosocial behavior, social-emotional learning, theory of mind

## Abstract

**Introduction:**

This study examines the effects of an integrated intervention combining inclusive children's literature and creative drama on the empathic tendencies of fourth-grade primary school students. Although the individual contributions of inclusive literature, creative drama, and empathy development have been documented separately in the literature, no prior study has examined how cognitive empathy initiated through inclusive narratives can be transformed into emotional and behavioral outcomes through drama-based experiences within a unified pedagogical framework. This study addresses that gap by both testing this theoretical proposition empirically and proposing an integrated model for multidimensional empathy education. Drawing on a multidimensional model of empathy (cognitive, affective, and behavioral) and grounded in developmental psychology and social-cognitive frameworks, particularly theory of mind and prosocial development, the study was conducted in the context of Turkish primary education, where inclusive policies are increasingly implemented yet remain insufficiently supported by evidence-based pedagogical models.

**Methods:**

A quasi-experimental pretest-posttest control group design was employed with 57 students enrolled in a public primary school in Türkiye. The experimental group participated in an eight-week structured intervention based on inclusive children's literature and creative drama, while the control group continued with regular classroom practices. As a methodological limitation, the intervention was implemented by different teachers across the experimental and control groups, which may have introduced teacher-specific variables that could not be fully controlled. Data were collected using the Empathic Tendency Scale, a validated instrument assessing multidimensional empathy constructs.

**Results:**

The results revealed that, after controlling for baseline scores, the experimental group demonstrated significantly higher posttest scores in overall empathic tendency compared to the control group, with a large effect size. Multivariate analyses further indicated that the intervention had significant effects across all subdimensions of empathy. While the effect on cognitive empathy was moderate, the effects on emotional and behavioral empathy were large, suggesting that the integration of drama-based experiential learning plays a critical role in transforming cognitive understanding into affective engagement and prosocial behaviors.

**Discussion:**

The results support the theoretical proposition that inclusive literature functions as a cognitive stimulus, whereas creative drama acts as a pedagogical mechanism that operationalizes empathy by embedding it in lived experience. By integrating inclusive education, children's literature, and creative drama within a unified framework, this study contributes to the literature by offering a novel and empirically tested model for fostering empathy in primary education. Future research should further examine the longitudinal stability of these effects, their transfer to real-life social behaviors, and their replicability across larger and more diverse samples.

## 1 Introduction

Empathy is one of the fundamental pillars of social and moral development, referring to the ability to understand others, share their feelings, and respond with helping behaviors. In educational contexts, fostering empathic skills is considered a key objective that supports children's social adjustment, academic achievement, and psychological well-being. Empathy is not a unitary construct but a multidimensional capacity encompassing cognitive, emotional, and behavioral components that develop in tandem across childhood (Davis, 1983). Cognitive empathy refers to the ability to take another's perspective and accurately infer their mental states. This capacity is closely linked to Theory of Mind development in developmental psychology (Kucirkova, 2019; Nikolajeva, 2016). Emotional empathy involves affective resonance with and sharing of another's inner experience (Hoffman, 2000). Behavioral empathy, in turn, represents the most concrete manifestation of this integration. It encompasses the translation of cognitive understanding and emotional resonance into prosocial actions such as helping, sharing, and cooperation (Davis, 1983; Liao, 2025). This tripartite structure has important implications for pedagogy. Interventions targeting only one dimension risk leaving the others underdeveloped. A child who intellectually understands another's feelings but remains emotionally unmoved, or who feels moved but fails to act, cannot be considered empathically competent in any full sense. Effective empathy education therefore requires approaches that address all three dimensions in an integrated and experiential manner.

Children's literature represents a well-established pathway into the cognitive dimension of empathy. Through fictional texts, children encounter characters whose inner lives differ from their own and engage in a form of “mind-reading,” using textual cues to infer characters' mental states, beliefs, and intentions (Kümmerling-Meibauer, 2014; Nikolajeva, 2016). A meta-analysis encompassing 30 studies demonstrated that story-based interventions yield moderate effect sizes on children's empathic skills, with adult-guided discussions during shared reading found to be particularly effective in supporting Theory of Mind development (Ciesielska et al., 2025; Mar et al., 2010). Inclusive children's literature deepens this engagement further by making individuals with diverse abilities, cultural backgrounds, and life experiences visible within literary texts. This opens space for children to view the world from multiple perspectives, challenge similarity bias, and engage in what Nikolajeva (2016) describes as indirect ethical experience – the rehearsal of moral reasoning through fictional situations. Inclusive representations have been shown to shape children's attitudes toward difference and promote awareness, understanding, and acceptance of individuals with diverse abilities (Alonge, 2024; Murphy, 2024; Prater et al., 2006; Sigmon et al., 2016), foster moral values such as respect and honesty, and increase prosocial behaviors such as sharing (Betawi, 2022). Yet a critical limitation must not be overlooked. The cognitive engagement initiated through reading does not readily translate into emotional resonance or behavioral change on its own. Literature creates the conditions for perspective-taking but does not inherently require children to embody, perform, or act upon what they have understood. It is at this point that creative drama assumes a critically important pedagogical function.

Creative drama activates the emotional and behavioral dimensions that literature alone largely leaves dormant. Its primary mechanism lies in the power of “as if” enactment. When a child assumes a role, they are afforded the opportunity to internalize the perspective, emotions, and motivations of that character rather than merely observing them from a cognitive distance (Wagner, 1976; Blair and Lutterbie, 2011). As Dorothy Heathcote's foundational approach emphasizes, putting oneself in another's shoes through drama is not a passive act of observation but an active, embodied experience (Edmiston and Towler-Evans, 2022; Heston, 2013). This embodied engagement has been associated with significant gains in social and emotional skills, reductions in aggression and victimization, and decreases in communication anxiety (Alaca et al., 2023; Corbett, 2019). From a neurobiological perspective, the mirror neuron system is argued to develop through such experiential engagement, thereby strengthening empathic capacity at a structural level (Keysers, 2011; Prentki, 2023). The structured stages of drama—preparation, enactment, and reflective evaluation—ensure that emotional experience does not remain transient. Mardas and Magos (2020) argue that meaning is truly constructed during the evaluation phase. This is the stage in which children discuss and cognitively process their emotional experiences. Drama thus transforms empathy from a cognitive achievement and a momentary feeling into a behaviorally consolidated capacity. This capacity is further made meaningful through reflective thinking. When drama activities are grounded in inclusive literary content, a still more far-reaching transformation occurs. The experience of difference encountered through narrative moves beyond abstraction and becomes a lived, embodied, and negotiated relational reality.

The setting in which this integrated process unfolds matters considerably. Inclusive educational environments bring together children with diverse abilities, backgrounds, and needs. They provide the social substrate within which empathy becomes not merely an academic exercise but a lived relational practice. Caballero (2024) found that students in inclusive classrooms develop more respectful, tolerant, and empathic attitudes through direct peer interaction. Qualitative evidence indicates that such contact heightens sensitivity and contributes to broader moral development. Ainscow (2020) similarly emphasized that inclusive education aims not only at supporting academic participation. It also seeks to cultivate the dispositions necessary for democratic and compassionate coexistence. In this context, inclusive children's literature goes beyond representing diversity at a merely symbolic level. As Bishop's (1990) metaphor of “mirrors, windows, and sliding glass doors” illustrates, books simultaneously serve as mirrors in which children recognize their own experiences and as windows that open onto the worlds of others (Baldwin, 2018). Drama-based activities can render this literary content experientially real for all learners, regardless of their backgrounds. The combination of inclusive narrative, performative embodiment, and socially heterogeneous learning environments therefore constitutes a theoretically coherent and pedagogically powerful framework for multidimensional empathy development.

Despite the robust findings within each individual body of literature, studies that integrate inclusive children's literature, creative drama, and empathy development all three within a single theoretically grounded intervention framework remain scarce. Existing research has examined the effects of drama on inclusivity (Sincuba and Mqondisi, 2025; Sun, 2025), the effects of literature on empathy and social-emotional development (Alonge, 2024; Ciesielska et al., 2025; Kucirkova, 2019; Kümmerling-Meibauer, 2014; Mar et al., 2010; Olmstead et al., 2023; Riquelme et al., 2013; Solow, 2018), the effects of empathy training on empathic tendencies toward peers with special needs (Elkoca and Bilgin, 2025), and the effects of drama on empathy (Alaca et al., 2023; Arda-Tuncdemir, 2025; Corbett, 2019; Mardas and Magos, 2020; Özyürek, 2021) largely in isolation or in pairwise combinations. To date, no study has examined how cognitive empathy initiated through fictional characters is transformed into emotional resonance and behavioral outcomes through structured drama activities. Beyond this primary gap, two further gaps in the existing literature merit attention. First, existing interventions have been conducted predominantly with early childhood or adolescent populations, leaving upper primary school—a developmental stage during which perspective-taking and prosocial reasoning are consolidating—comparatively underexplored (Hoffman, 2000; Piaget, 1952). Second, and perhaps most critically, few studies have been conducted in genuinely inclusive classroom settings that bring together students with special educational needs, students with migration backgrounds, and typically developing peers simultaneously. Yet it is precisely in such heterogeneous environments that empathy education is both most pedagogically challenging and most urgently needed. Compounding these gaps, existing interventions have rarely evaluated empathic development using instruments that simultaneously measure all three dimensions. The developmental trajectory across cognitive, emotional, and behavioral planes therefore remains insufficiently understood. This collective absence is not merely methodological. It reflects a broader lack of integrated pedagogical models that operationalize empathy as a multidimensional and sequentially developing construct within educational practice.

The present study aims to address this gap by empirically testing the effects of an intervention model that integrates inclusive children's literature with creative drama on the empathic tendencies of fourth-grade primary school students. The intervention is grounded in Davis's (1983) multidimensional model of empathy and Vygotsky's (1978) sociocultural framework. Vygotsky's concept of the Zone of Proximal Development is operationalized within the study specifically in the context of drama-based role enactment (Metinnam, 2025). Within this theoretical foundation, inclusive narratives initiate cognitive perspective-taking, which is subsequently transformed through structured drama activities first into emotional resonance and then into behavioral response. Eight thematic categories of inclusive content bring students into contact with a range of experiences of “otherness,” while the preparation–enactment–evaluation structure of each drama session ensures that literary comprehension is translated into embodied experience and reflective understanding. By examining the effects of this integrated approach on both overall empathic tendencies and each empathic dimension separately, the study aims to contribute to the literature a theoretically coherent, empirically tested model for multidimensional empathy education in inclusive primary school contexts. The following research questions guided the study:

- Does the drama-based inclusive children's literature intervention have a significant effect on students' overall empathic tendencies compared to the control group?
- Does the intervention have a significant effect on students' cognitive empathy levels?
- Does the intervention have a significant effect on students' emotional empathy levels?
- Does the intervention have a significant effect on students' behavioral empathy levels?

## 2 Theoretical framework

### 2.1 Concept of empathy and its multidimensional structure

Empathy is regarded as one of the most fundamental components of human social interaction and moral development and is defined as the capacity to understand another individual's inner world, emotions, intentions, and perspectives, and to respond appropriately to this understanding. Originating from the German term Einfühlung (feeling into), the concept of empathy has been discussed across a wide range of disciplines from philosophy to psychology; however, in contemporary literature, it is conceptualized not as a unidimensional construct but as a dynamic and functional interaction of cognitive, emotional, and behavioral components (Cuff et al., 2016; Wispé, 1986; Davis, 1983). In inclusive educational settings, particularly in fostering positive attitudes toward disadvantaged groups, individuals with special needs, and peers from diverse cultural backgrounds, each component of this multidimensional structure plays a critical and complementary role (Ainscow, 2020; Kucirkova, 2019). Accordingly, in the present study, empathy is conceptualized as a process that is cognitively initiated through inclusive children's literature and subsequently transformed into emotional and behavioral dimensions through creative drama.

Cognitive empathy is defined as the ability to take another person's perspective (perspective-taking) and to accurately infer their mental states, beliefs, and intentions. This dimension is closely and inseparably related to the concept of Theory of Mind in developmental psychology (Kucirkova, 2019). Theory of Mind refers to the ability to understand that others may possess knowledge, beliefs, and desires different from one's own, and therefore may perceive the world differently (Nikolajeva, 2016). For a primary school child, cognitive empathy goes beyond merely recognizing that a peer is upset; it involves making rational inferences about why that peer might feel that way and interpreting their mental state. For example, when a student reads about a character in inclusive children's literature (e.g., a child with dyslexia or a migrant background) and analyzes the reasons behind that character's behavior from their perspective, the mechanism of cognitive empathy is activated (Alonge, 2024; Ciesielska et al., 2025; Kümmerling-Meibauer, 2014). In this process, the child mentally simulates the character's perspective, moves beyond their own experiential framework, and cognitively makes sense of differences (Ciesielska et al., 2025; Mar and Oatley, 2008; Orvell et al., 2019). Research indicates that cognitive empathy training through children's literature, particularly via engagement with diverse characters, reduces prejudice and enhances social cognition (Kucirkova, 2019).

Emotional empathy, on the other hand, refers to the direct sharing of another person's emotional state, emotional resonance, and the tendency to respond with similar affective reactions. Unlike cognitive empathy, this process is primarily grounded in affective experience, co-feeling, and emotional contagion (Davis, 1983; Hoffman, 2000; Mardas and Magos, 2020). Emotional empathy enables individuals to internally experience others' joy or distress, thereby fostering a sense of connectedness and strengthening social bonds. However, it has been noted that emotional empathy alone may not be sufficient; when not balanced by cognitive processes, it may lead to empathic distress or emotional exhaustion (Cuff et al., 2016; Krasner et al., 2009; Özdelikara and Babur, 2020; Swan and Riley, 2015). As emphasized in Dorothy Heathcote's drama approach, when a child “puts themselves in someone else's shoes,” they do not merely observe another person's emotional world but actively experience it (Heston, 2013; Wagner, 1976; Edmiston and Towler-Evans, 2022). This experiential process transforms emotional empathy from a transient feeling into a more enduring sensitivity. Emotional empathy developed through children's literature and drama enhances children's emotional literacy and supports their social-emotional learning (Corbett, 2019; Solow, 2018; Olmstead et al., 2023).

The transformation of empathic understanding and emotional sharing into prosocial actions such as helping or supporting others is defined as behavioral empathy. This dimension represents the most concrete and operationalized manifestation of the integration of cognitive understanding and emotional resonance (Davis, 1983; Liao, 2025). In the present study, behavioral empathy is conceptualized as the observable outcomes of empathic tendencies. Prosocial behaviors encompass a wide range of actions, including sharing, cooperation, social participation, and responsiveness to others' distress (Bhavnagri and Samuels, 1996). Berndt (1983) emphasized that strategies such as sharing and helping, fostered through children's literature, play a fundamental role in the development of peer relationships.

Research has shown that children with higher levels of empathic tendencies display more accepting attitudes toward peers with special needs in inclusive classroom settings. Such students tend to develop respect for diversity and contribute to a more positive classroom climate (Caballero, 2024). As a central mechanism in social meaning-making, empathy also serves as an important protective factor against antisocial behaviors (Jolliffe and Farrington, 2004; Lawrence et al., 2004). Furthermore, Betawi (2022) argued that empathy is closely associated with moral values such as honesty, respect, and courage.

Children's literature plays a central role in transforming empathic abilities into concrete actions. Shared reading processes, particularly those involving discussions about characters' intentions and emotions with adults, facilitate children's understanding of others' mental states (Mar et al., 2010). While personalized stories have been shown to produce significant increases in sharing behaviors (Kruse et al., 2020), Kumschick et al. (2014) emphasized that literature-based interventions help children understand characters' motivations and develop emotional competence.

According to Piaget's (1952) theory of cognitive development, the fourth-grade level (ages 9–10) represents a transitional phase from the concrete operational stage to the formal operational stage. During this period, egocentrism decreases, and children begin to develop the ability to take others' perspectives. Hoffman (2000) suggested that prosocial moral development at this stage shifts from externally imposed rules to an internalized sense of responsibility. It has also been noted that moral narratives that emphasize positive outcomes promote honest behavior (Liang et al., 2025). This developmental shift in empathy enables children to extend their sense of responsibility beyond their in-group to include individuals from diverse backgrounds, such as those with disabilities or migrant experiences (Kucirkova, 2019).

In conclusion, the cognitive, emotional, and behavioral dimensions of empathy function together to shape individuals' social intelligence and moral development. The integrated interaction of these dimensions constitutes the primary theoretical foundation of the present study. When combined with inclusive texts and drama-based experiences, the developmental potential of fourth-grade students can transform empathy into an operational and observable construct within educational practice.

### 2.2 Inclusive children's literature: representation of diversity and literary tools

In this study, inclusive children's literature is conceptualized as a domain that makes visible individuals with diverse abilities, cultural backgrounds, and life experiences within literary texts, representing their stories and achievements in authentic and meaningful ways. In explaining the role of multicultural children's literature, Alonge (2024) argues that such works provide children with opportunities to move beyond the boundaries of their own experiences and to understand others' lives, values, and emotions. Engagement with rich and diverse picture books strengthens empathic abilities by enabling children to both observe and imagine others' experiences (Chen and Browne, 2015; Souto-Manning, 2009; Williams-Sanchez, 2021).

Themes such as visual impairment, dyslexia, ADHD, migration, and socioeconomic differences offer children the opportunity to view the world from multiple perspectives. When children identify with characters who share similar traits or cultural backgrounds, they develop a sense of validation and belonging (Alonge, 2024). Kurtts and Gavigan (2008) suggest that children's books provide important insights into the lives of individuals with disabilities and that reading about others' struggles fosters sensitivity. In this respect, the critical role of inclusive representations in enhancing children's empathy and understanding has been emphasized (Murphy, 2024).

Indirect contact established through literature serves as a key mechanism by which children's engagement with characters can reduce real-life prejudice. Nikolajeva (2016) states that fictional texts create a form of indirect ethical experience by placing characters in situations where ethical dilemmas are inevitable, thereby making such issues more concrete and comprehensible. Through “narrative empathy,” prejudices can be reduced, and particularly in multicultural societies, “similarity bias” can be challenged through these texts (Hoffman, 2000). In this context, inclusive literature functions as a starting point that primarily activates cognitive empathy.

The reading process can be described as a form of “mind-reading,” in which readers engage in mental simulation by using textual cues to understand characters' mental states. During this process, readers draw upon their own world knowledge to make inferences about characters' inner worlds (Kümmerling-Meibauer, 2014). Studies have shown that discussions between children and their parents about books positively contribute to the development of Theory of Mind skills (Mar et al., 2010). Similarly, Olmstead et al. (2023) argue that educators can incorporate such inclusive content into curricula to provide children with authentic cultural perspectives.

Identification with characters and perspective-taking constitute the primary mechanisms through which inclusive literature fosters cognitive empathy. Although effective mind-reading requires a certain degree of distance from the character's perspective (Nikolajeva, 2016), this does not prevent readers from adopting that perspective. Kruse et al. (2020) demonstrated that the degree to which children relate to characters directly influences their learning processes and behavioral changes. This interaction with fictional texts is considered an important indicator of the development of social cognition, including empathy (Kümmerling-Meibauer, 2014).

The role of inclusive literature in moral development lies in its capacity to support children in constructing confident identities, engaging fairly with differences, and developing critical thinking skills in response to injustice. This approach introduces children to concepts such as justice and equality (Derman-Sparks and Task Force, 1989). Inclusive education, which aims to reduce barriers to participation for all children, can be effectively supported through inclusive children's literature (Booth and Ainscow, 2002).

When literature is combined with methods such as creative drama, the scope of empathy expands beyond human interactions to include all living beings in nature. (Özyürek 2021) found that drama-based instructional practices increase individuals' awareness of empathizing not only with humans but also with animals and plants. This suggests that the combination of inclusive literature and drama broadens the boundaries of empathy. This function is well illustrated by Bishop's (1990) metaphor of “mirrors, windows, and sliding glass doors,” which describes books as tools that both reflect children's own experiences and provide windows into the humanity of others (Baldwin, 2018).

In conclusion, inclusive children's literature teaches children to perceive diversity as a richness and to critically reflect on their existing prejudices. As summarized by Alonge (2024), through the combined effects of empathy and cultural awareness, multicultural children's literature plays a critical role in raising a generation that values inclusivity and respects all individuals. The eight thematic categories employed in this study introduce children to a range of “otherness” experiences, thereby enhancing their empathic capacity, social cognition, and moral reasoning. These literature-based interventions enable children to internalize inclusivity not only at the cognitive level but also at emotional and behavioral levels.

### 2.3 Creative drama as a method and its relationship with empathy

Creative drama provides an experiential learning environment that supports children's empathic development processes. San (1996) defines this discipline as the process through which individuals reconstruct their existing cognitive schemas and make sense of and enact an event, idea, educational unit, or concept through playful processes. What distinguishes this process from theater is its emphasis on sharing, collaborative creativity, and collective production (San, 2006).

(Üstündag 1998), emphasizing the importance of transforming students from passive listeners into active participants through the engagement of the body and senses, argues that learning content should be made experiential through enactment. Similarly, Arda-Tuncdemir (2025) states that creative drama activities provide students with opportunities to interact with one another and with materials, thereby expanding both their social relationships and knowledge base. Through this experiential process, empathy—an otherwise abstract concept—becomes concrete; rather than receiving theoretical instruction about empathy, children directly experience it by taking on the role of another person. The outcomes facilitated by drama also contribute significantly to the development of critical thinking, moral reasoning, and a broader worldview (Mardas and Magos, 2020). Furthermore, these processes are reported to foster essential perspective-taking skills associated with empathy (Corbett, 2019).

The primary mechanism underlying the role of creative drama in empathic development lies in the power of “as if” enactment. When a child assumes a role, they are afforded the opportunity to internalize the perspective, emotions, and motivations of that character (Wagner, 1976). Blair and Lutterbie (2011) emphasize that students participating in drama activities gain opportunities to approach and understand the thoughts and actions of fictional characters. This pedagogical approach situates both classroom communities and the explored topics within a more humanized framework (Edmiston and Towler-Evans, 2022).

The structured stages of drama play a critical role in ensuring the effectiveness of the process. During the warm-up phase, participants are psychologically prepared to enter roles through the activation of the five senses and the development of a sense of trust. In the enactment phase, a creative process unfolds in which children, within a framework of established rules, freely construct and develop scenarios through techniques such as pantomime and role-playing (Tuluk, 2004). However, Mardas and Magos (2020) argue that action alone is not sufficient and that the reflective thinking and evaluation phase following enactment is where meaning is truly constructed. These reflective discussions allow children to cognitively process their emotional experiences.

Prentki (2023) emphasizes that teachers should be trained as facilitators in the development of civility and highlights that drama provides unique opportunities for practicing critical empathy skills. Discussions during the evaluation phase enable children to consider not only their own experiences but also alternative perspectives through feedback on their peers' improvisations (Alaca et al., 2023). Developed by pioneers such as Dorothy Heathcote and Peter Slade in the twentieth century, drama has come to be recognized as a systematic teaching and learning method (Mukhibova et al., 2022; Wagner, 1976). Establishing a safe learning environment is essential for enabling children to take risks and engage in new empathic experiences. This supportive space, created by teachers who attend to each student's unique cultural background and needs, reinforces a sense of openness and trust (Arda-Tuncdemir, 2025). Metinnam (2025), drawing on Vygotsky's concept of the Zone of Proximal Development, explains that when teachers enter roles within drama, they create a dynamic rehearsal space in which students can experiment with social identities, ethical choices, and democratic participation.

From a neurobiological perspective, the mirror neuron system in the human brain can develop through experiences that reshape how individuals perceive others' actions, thereby enhancing empathic capacity (Keysers, 2011). Emphasizing that this development is a collective process, Heston (2013) highlights the importance of egalitarian and participatory structures—such as forming a circle with children—rather than adhering to traditional classroom arrangements. In conclusion, creative drama sessions that incorporate preparation, enactment, and evaluation phases provide children with opportunities to experience empathy, feel emotional intensity, and consolidate these experiences through reflective thinking. This holistic impact of drama can simultaneously develop the cognitive, emotional, and behavioral dimensions of empathy, transforming it from a theoretical construct into an integral part of children's lived experience.

### 2.4 Synthesizing inclusive literature and drama: operationalizing empathy

The integration of inclusive children's literature and creative drama enables empathy to move beyond a purely cognitive disposition and to become a holistic process experienced through emotional and behavioral dimensions. In this sense, empathy is transformed not only into a mental process of understanding but also into an observable and experiential learning outcome. Nikolajeva (2016) emphasizes that cognitive engagement with fictional texts constitutes a bidirectional process that extends from life to text and from text back to life. Within this framework, inclusive literature provides the conceptual foundation necessary for perspective-taking by introducing children to diverse lived experiences through literary characters.

Creative drama, in turn, transforms this theoretical foundation into concrete action. During drama activities, students have been observed to enthusiastically fill gaps in understanding, engage in dynamic discussions, and express their emotions while demonstrating a willingness to understand others' life conditions (Mardas and Magos, 2020). In this process, the child not only cognitively comprehends the character but also embodies the role, experiencing the situation directly at both bodily and emotional levels. (Kümmerling-Meibauer 2014) argues that one of the primary functions of fictional narratives is to evoke and regulate empathy in the reader. This emotional engagement is further deepened through drama, as inclusive content is enacted and embodied through techniques such as role-playing and improvisation. Thus, the mental simulation initiated through reading is transformed into an experiential learning process.

**Figure 1:**
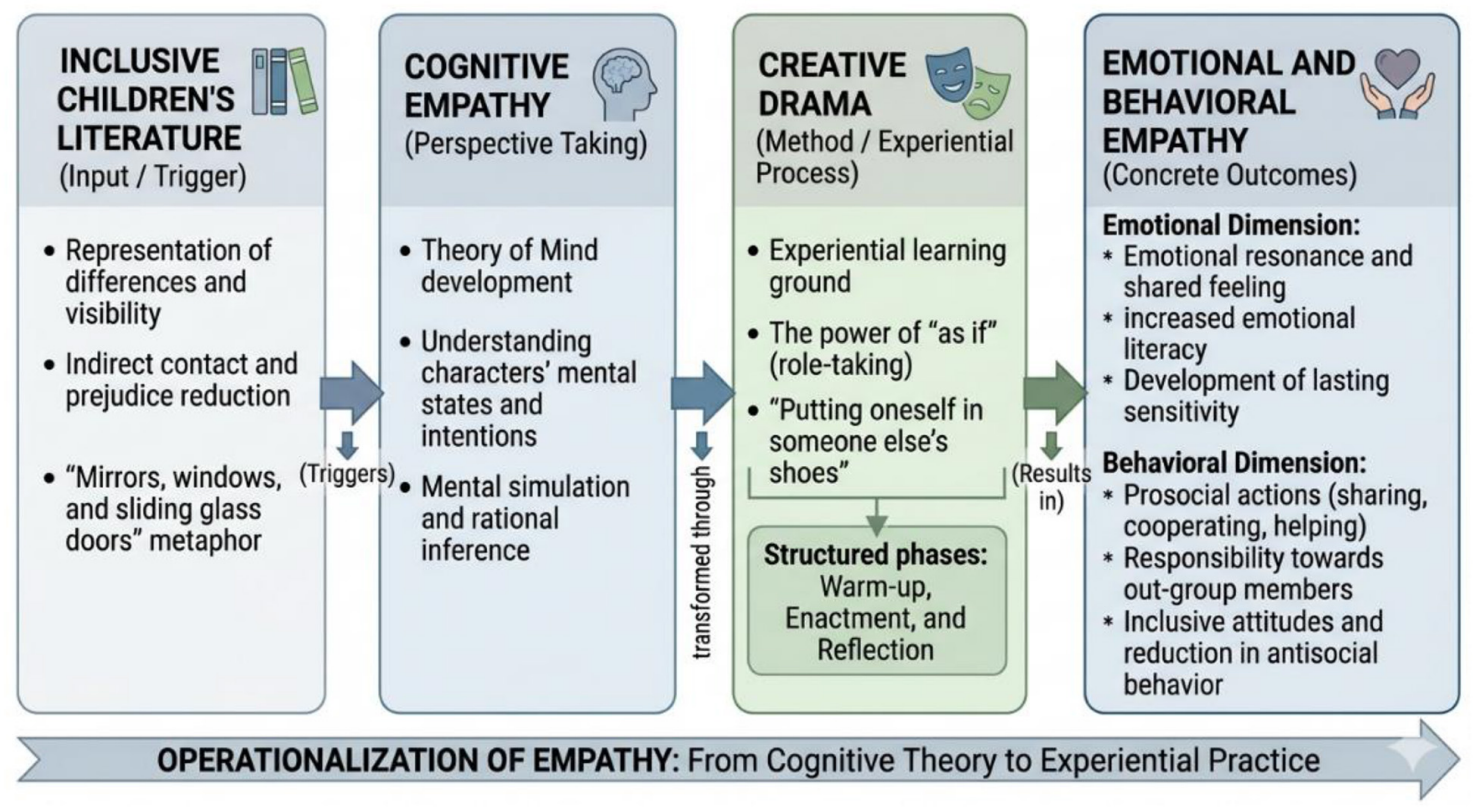
Conceptual model of operationalizing empathy through inclusive children's literature and creative drama.

In conclusion, within the framework of this study, inclusive children's literature is positioned as a cognitive stimulus and a source of meaning for empathy, while creative drama functions as a pedagogical tool that concretizes this cognitive process at emotional and behavioral levels. The integration of these two approaches enables empathic tendencies to evolve from a purely theoretical construct into observable and measurable learning outcomes through pedagogical processes. The conceptual framework proposed in this study, which explains how empathy is transformed into cognitive, emotional, and behavioral dimensions through the interaction of inclusive children's literature and creative drama, is presented in Figure 1.

As illustrated in Figure 1, empathy is conceptualized as a process that is cognitively initiated through literature and experientially transformed into emotional and behavioral outcomes through drama.

## 3 Method

### 3.1 Research model

This study employed a quasi-experimental pretest–posttest control group design. Such designs are widely used in educational research when random assignment is not feasible, and intact classrooms must be used as naturally existing groups (Creswell, 2012). In the present study, one classroom was designated as the experimental group and received the structured drama-based inclusive education intervention, while the other classroom served as the control group and continued with standard Free Activities practices. Both groups were administered pretest and posttest measures to examine changes over time and differences attributable to the intervention.

### 3.2 Study group

The population of the study consisted of 147 fourth-grade students enrolled in a public primary school in Bursa, Türkiye, during the 2025–2026 academic year. The sampling process was conducted in two stages. First, the school was selected using convenience sampling based on accessibility and feasibility considerations. In the second stage, criterion sampling, a type of purposive sampling, was employed for class selection. Classrooms that included students within inclusive education settings (e.g., students with special educational needs and/or migration backgrounds) were identified, and those with a relatively higher representation of such students were preferred. This approach enabled the study to be conducted in naturally diverse classroom environments reflecting varying learning needs and socio-cultural backgrounds. Following the selection of these classrooms, participants were assigned to the experimental and control groups based on existing classroom structures. No artificial grouping was introduced, and the natural classroom context was preserved in order to maintain ecological validity.

To determine the adequacy of the sample size, a power analysis was conducted using G^*^Power 3.1. Given that the study employed an ANCOVA design controlling for pretest scores, the analysis was performed using the *F*-test family with the “linear multiple regression: fixed model, *R*^2^ increase” option. The effect size was derived from the study data; the partial eta squared value for the group effect (η^2^ = 0.19) was converted into Cohen's *f*^2^ (*f*^2^ = 0.23). With an alpha level of 0.05, a statistical power of 0.95, one tested predictor (group), and two total predictors (group and pretest scores), the minimum required sample size was estimated as 59 participants.

At the beginning of the study, 60 students were included. However, three participants (two from the experimental group and one from the control group) were excluded from the analysis due to their absence from the intervention sessions for 4 weeks. As a result, the study was completed with 57 students (28 in the experimental group and 29 in the control group). Although the final sample size fell slightly below the estimated minimum, the difference was minimal and can be regarded as acceptable within school-based research contexts, where attrition is relatively common. To further examine the potential impact of this shortfall, a *post-hoc* sensitivity analysis was conducted using G^*^Power 3.1. With *N* = 57, α = 0.05, and the obtained effect size (*f*^2^ = 0.23), the achieved power was approximately 0.94, exceeding the conventional 0.80 threshold (Cohen, 1988). While *post-hoc* power estimates have well-recognized limitations and should therefore be interpreted cautiously, this result provides supportive evidence that the reduction in sample size was unlikely to have substantially influenced the study's ability to detect intervention effects.

Beyond statistical considerations, the decision to retain intact classroom structures rather than recruit additional participants was informed by the pedagogical nature of creative drama. Drama-based learning is more effective in small, stable, and interactive groups that support active participation and sustained peer interaction (McCaslin, 2006; Selvi, 2003). Selvi (1999) further notes that overcrowded classrooms may reduce the effectiveness of drama activities and recommends limiting group size to approximately 25 students. Accordingly, increasing the sample would have required merging classrooms or altering existing group structures, potentially compromising interaction quality and instructional authenticity. Thus, the final sample reflects a balance between statistical adequacy and pedagogical integrity.

This rationale is also consistent with the broader literature. Previous studies on drama-based instruction in primary education have typically used relatively small, intact classroom samples. For example, studies involving fourth-grade students have reported sample sizes ranging from approximately 36 to 54 participants (Cer, 2017; Rew and Moon, 2013; Seven, 2021). Overall, these findings suggest that drama-based educational research is generally conducted within natural classroom settings rather than large, externally constructed samples.

The demographic and contextual characteristics of the study group are presented in Table 1 to provide a clearer overview of the participants' profiles.

**Table 1:** Descriptive characteristics of the study group.

Variable	Category	Experimental	Control	Total
Gender	Girls	13 (46.4%)	14 (48.3%)	27 (47.4%)
	Boys	15 (53.6%)	15 (51.7%)	30 (52.6%)
	Total	28 (100%)	29 (100%)	57 (100%)
Inclusive education status	Inclusion student	1	1	2
	– Learning disability	1	–	1
	– Mild intellectual disability	–	1	1
Migration background	Migrant students	2	2	4
	– Syria	2	1	3
	– Afghanistan	–	1	1

According to Table 1, the study group consisted of 57 fourth-grade students, of whom 27 (47.4%) were girls and 30 (52.6%) were boys. The experimental group comprised 13 girls (46.4%) and 15 boys (53.6%), whereas the control group included 14 girls (48.3%) and 15 boys (51.7%). The gender distribution was comparable across groups.

Two students received inclusive education services. These students had medical diagnoses issued by authorized healthcare institutions and educational evaluation reports provided by Guidance and Research Centers (RAM). The student in the experimental group had been diagnosed with a learning disability since the second grade, while the student in the control group had been identified with a mild intellectual disability since preschool. Both students were girls and received 6 h of weekly support in a resource room in accordance with their individualized education plans (IEPs). Both had prior special education histories predating their enrollment in fourth grade.

Four students had a migration background—three from Syria and one from Afghanistan. Of the two migrant students in the experimental group, one was a girl and one was a boy (both from Syria). In the control group, one student was a girl from Syria and the other was a boy from Afghanistan. All four students were born in Türkiye to families who had immigrated due to war and conflict in their countries of origin. Within their families, they acquired Turkish bilingually alongside their heritage languages. By the fourth grade, their Turkish proficiency had been consolidated through approximately 5 years of schooling in the Turkish education system, and all four students demonstrated age-appropriate Turkish language skills at the time of the study.

Preliminary analyses indicated that the experimental and control groups did not differ significantly at baseline in terms of cognitive, emotional, or behavioral empathic tendency scores, suggesting group equivalence prior to the intervention.

All participants were enrolled in the same public school and followed the same national curriculum, ensuring comparable instructional conditions. The school is located in an urban area and serves students from diverse socio-economic backgrounds. In addition, the presence of students with special educational needs and migrant backgrounds in both groups suggests that the study group was not homogeneous, but rather represented a naturally inclusive classroom structure.

Participation in the study was voluntary. Prior to data collection, informed consent was obtained from the parents or legal guardians of all participants. Students were also informed about the study in an age-appropriate manner, and their verbal assent was obtained. Necessary permissions were secured from the relevant institutional authorities. The experimental group received the inclusive drama-based children's literature intervention, while the control group continued with the regular curriculum.

### 3.3 Data collection instrument

In this study, the Empathic Tendency Scale developed by (Kilinç and Sözer 2022) was used to determine students' levels of empathic tendency. The scale was developed for primary school students and consists of 17 items organized under three dimensions: cognitive empathy, emotional empathy, and behavioral empathy. It is a four-point Likert-type instrument (1 = Never, 4 = Always). The internal consistency coefficient (Cronbach's alpha) for the overall scale was reported as 0.88, indicating satisfactory reliability. These findings suggest that the Empathic Tendency Scale is a valid and reliable instrument for assessing empathic tendencies among primary school students.

In the present study, internal consistency coefficients were calculated separately for the pre-test and post-test administrations to examine the reliability of the scale within the current sample. Cronbach's alpha for the overall scale was 0.84 in the pre-test and 0.90 in the post-test, indicating good to excellent internal consistency. Corrected item-total correlations ranged between 0.29 and 0.64 for the pre-test and between 0.39 and 0.74 for the post-test. No items were removed, as deleting any item did not result in a meaningful increase in the reliability coefficient, supporting the retention of the original 17-item structure.

For the sub-dimensions, alpha coefficients ranged from 0.58 to 0.74 in the pre-test and from 0.72 to 0.84 in the post-test. While the behavioral empathy subscale yielded a relatively lower internal consistency in the pre-test (α = 0.58), this may be attributed to the limited number of items within the subscale and the developmental characteristics of the participants. Importantly, item analyses demonstrated that removing any item did not substantially improve reliability, suggesting that the dimensional structure remained stable. In the post-test, reliability coefficients for all sub-dimensions exceeded the acceptable threshold (α ≥ 0.70), indicating improved internal consistency following the intervention.

Overall, these findings provide evidence that the scale demonstrated satisfactory psychometric properties in the present study and maintained its multidimensional structure across measurement occasions.

### 3.4 Data analysis

Descriptive statistics, including mean, standard deviation, median, variance, minimum–maximum values, skewness, and kurtosis, were computed to examine the distribution of the data. Skewness and kurtosis values for all dependent variables fell within the ±1 range, indicating that the distributions were approximately normal (e.g., total empathic tendency: skew = −0.52, kurtosis = −0.13; cognitive empathy tendency: skew = −0.44, kurtosis = −0.71). Homogeneity of variance was verified using Levene's test, confirming that the assumption was met for all dependent variables (*p* > 0.05). Pearson correlations among the dependent variables ranged from 0.421 to 0.935, demonstrating adequate relationships between subdimensions. No outliers were observed.

Baseline equivalence between the experimental and control groups was assessed using independent samples *t*-tests, which indicated no significant differences across any dependent variables (*p* < 0.05), confirming that the groups were comparable prior to the intervention.

To evaluate the effect of the drama-based inclusive children's literature intervention on students' total empathic tendency, an analysis of covariance (ANCOVA) was conducted while controlling for pretest total scores. Subsequently, a multivariate analysis of covariance (MANCOVA) was performed to assess the intervention's impact on the subdimensions of empathic tendency (cognitive, emotional, and behavioral), controlling for their respective pretest scores. This approach allowed for a comprehensive examination of the intervention's effects on both overall empathic tendency and its specific subdimensions.

### 3.5 Intervention procedure

This study was conducted during the 2025–2026 academic year in a public primary school in Bursa using a quasi-experimental design. Prior to data collection, institutional ethics committee approval and official permission from the Ministry of National Education (MoNE) were obtained. Preliminary meetings were held with the school administration and classroom teachers before the implementation phase. Among the five fourth-grade classrooms in the school, two were selected through criterion sampling as inclusive classrooms reflecting diverse learning characteristics and needs. The teachers of these classrooms were informed about the study procedures and agreed to participate voluntarily. The two intact classrooms were then randomly assigned to the experimental and control conditions.

Each classroom was taught exclusively by its regular classroom teacher throughout the 8-week intervention period, with no teacher exchange between conditions. Both teachers had comparable professional experience exceeding 10 years and worked within the same school context and curriculum framework. Although both teachers had prior experience with student-centered instructional approaches, including drama-based activities, neither had previously implemented a structured drama-based intervention of the type used in the present study. To reduce potential teacher-related variability, the researcher prepared detailed weekly drama lesson plans for the experimental group and shared them in advance with the responsible teacher. All instructional materials were provided prior to implementation, and implementation fidelity was supported through ongoing monitoring.

The intervention was conducted during the officially designated “Free Activities” course hours, which, according to national curriculum guidelines, are intended to support students' personal and social development through activities such as reading, drama, film viewing, games, excursions, sports, and artistic engagement, taking into account students' age and developmental levels. In this context, the drama-based inclusive education program was implemented as a structured enrichment aligned with the course objectives.

Both groups participated in Free Activities lessons during the allocated time; however, the nature of these activities differed between conditions. The experimental group received a structured, theoretically grounded, and sequential drama-based inclusive education program integrating literary content to foster inclusion and empathy. In contrast, the control group continued with routine classroom practices determined by the teacher, including activities such as silent reading, educational games, drawing, short film viewing, and unstructured group tasks. No structured drama-based empathy program or systematic inclusion-focused literary sequence was introduced in the control group.

The decision not to implement an alternative structured program in the control group was methodologically grounded in the quasi-experimental design of the study. This approach enabled a comparison between the effects of a systematically designed intervention and the naturally occurring Free Activities practices within the school context, thereby allowing the assessment of the added value of the drama-based inclusive education program. Accordingly, while both groups engaged in developmentally appropriate Free Activities sessions, only the experimental group participated in a structured and literature-integrated intervention. This distinction ensured ecological validity while preserving internal validity for the analysis of intervention effects.

The theoretical foundation of this design is grounded in the function of literature-based approaches within inclusive education. Such approaches facilitate the understanding of differences by providing developmentally appropriate narratives for primary school students. In particular, previous research has emphasized that the use of picture books with primary school children constitutes a developmentally appropriate and effective instructional tool (Prater et al., 2006; Sigmon et al., 2016). Within this framework, the use of children's literature was structured by considering that the fourth-grade students who constituted the research group display characteristics of the transition from the concrete operational stage to the formal operational stage (Piaget, 1972). During this period, children are assumed to develop skills related to interpreting concrete events, analyzing narrative structures, understanding abstract concepts through concrete examples, and acquiring basic perspective-taking abilities. Furthermore, in line with Vygotsky's (1978) sociocultural theory, it was assumed that guided interaction and scaffolding processes support meaning-making through shared reading and dramatization activities.

The literary component of the intervention consisted of a combination of printed children's books and digital resources. Printed materials were selected from publications of the Scientific and Technological Research Council of Türkiye (TÜBITAK), whereas digital materials were obtained from the MoNE. These institutions were specifically preferred because they are recognized as reliable and authoritative providers of educational content in Türkiye and ensure both quality and curriculum alignment.

Book selection was conducted through a systematic process. In the first stage, a pool of 28 children's books was created from MoNE and TÜBİTAK publications. All books focused on inclusive education themes. The books were then evaluated according to the following criteria: developmental appropriateness for fourth-grade students, thematic relevance to inclusive education, narrative depth supporting perspective-taking, and suitability for dramatization. To determine the appropriateness of the materials, expert opinions were obtained from three independent reviewers: a primary school teacher, a special education teacher, and an academic specializing in children's literature. No formal rating scale was administered during the selection process; however, expert evaluations were informed by established quality criteria regarding the representation of disability in children's literature. In this respect, selected principles from the Rating Scale for Quality Characterizations of Individuals with Disabilities in Children's Literature, originally developed by Dyches and Prater (2000, 2005) and later revised and expanded by Taylor and Prater (2020), were used as a conceptual guide. In particular, experts considered the scale's key dimensions (social interactions, exemplary practices, point of view, literary quality of text, and illustrations) when reviewing candidate books. The selected books were also subjected to a pilot read-aloud session in a fourth-grade classroom that was not included in the intervention group. This pilot implementation was conducted to examine comprehensibility, student engagement, and suitability for the planned session duration.

**Figure 2:**
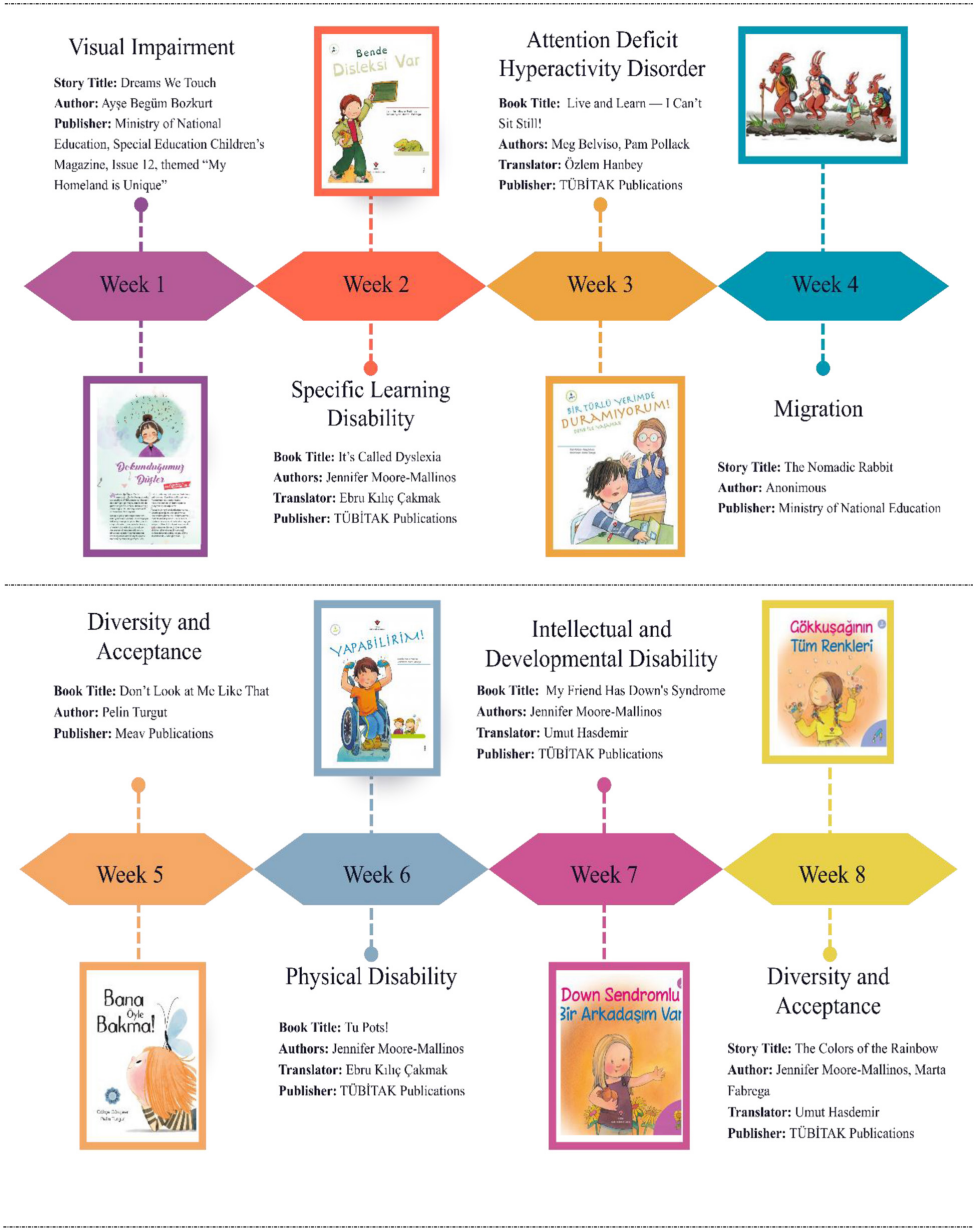
Weekly schedule of books used in the intervention.

As a result, each selected children's book represented a different dimension of inclusion, including visual impairment, specific learning disability (dyslexia), attention deficit and hyperactivity disorder (ADHD), migration, physical disability, intellectual and developmental disability, and diversity and acceptance. This process was further supported by Mayer's (2009) multimedia learning principles, enabling students to access content through both print and digital modalities. This multimodal presentation enhanced accessibility for learners with diverse learning profiles and strengthened students' readiness to engage with literary tools. The weekly thematic distribution and corresponding literary texts are presented in Figure 2.

The selected children's books were used not merely as reading materials, but as components of a structured text-processing sequence integrated into each intervention session. In each weekly session, work with the literary texts followed a holistic progression consisting of pre-reading preparation, interactive comprehension, dramatic enactment, and reflective evaluation. The comprehension phase of the intervention was informed by established instructional recommendations regarding the use of children's literature in inclusive classrooms. In line with Prater et al. (2006), prereading strategies were implemented to direct students' attention to the actions, emotions, and experiences of characters within the narrative. These strategies included brief discussions, prediction prompts, key-concept exploration, and theme-related warm-up tasks aimed at activating prior knowledge and supporting students' cognitive and emotional readiness for the text. During the comprehension phase, the primary school teacher conducted a model read-aloud while the text and illustrations were simultaneously projected onto the smart board so that students could follow the story visually in real time. Consistent with Prater et al. (2006), students were first encouraged to engage with the text without interruption in order to construct holistic meaning. Subsequently, at predetermined points, the teacher paused to invite predictions, discuss characters' emotions, interpret illustrations, and examine cause-and-effect relationships within the narrative. This second reading phase enabled more detailed interpretation and reflective discussion, particularly focusing on characters representing diverse learning and social needs. In this way, the books functioned as interactive meaning-making tools rather than passive listening materials. During the meaning-extension and self-expression phases, students related the inclusion themes presented in the stories to their own experiences, discussed the perspectives of the characters, and subsequently reconstructed the narratives through creative drama techniques such as role-play, improvisation, frozen image, and inner voice. This process enabled students to move from cognitive understanding of the story toward embodied and affective engagement. Finally, the evaluation phase focused on guided reflection regarding the difficulties experienced by the characters, the emotions involved, and ways of offering support in real-life situations. Thus, the text-processing sequence functioned as a structured pedagogical cycle designed to foster empathic awareness and inclusive attitudes.

Each weekly session lasted approximately 80 min and followed a consistent drama-based sequence consisting of experiential preparation, narrative engagement, dramatic enactment, and structured reflection. The recurrence of this sequence ensured procedural fidelity, while thematic variation across weeks broadened students' exposure to diverse forms of social difference.

**Figure 3:**
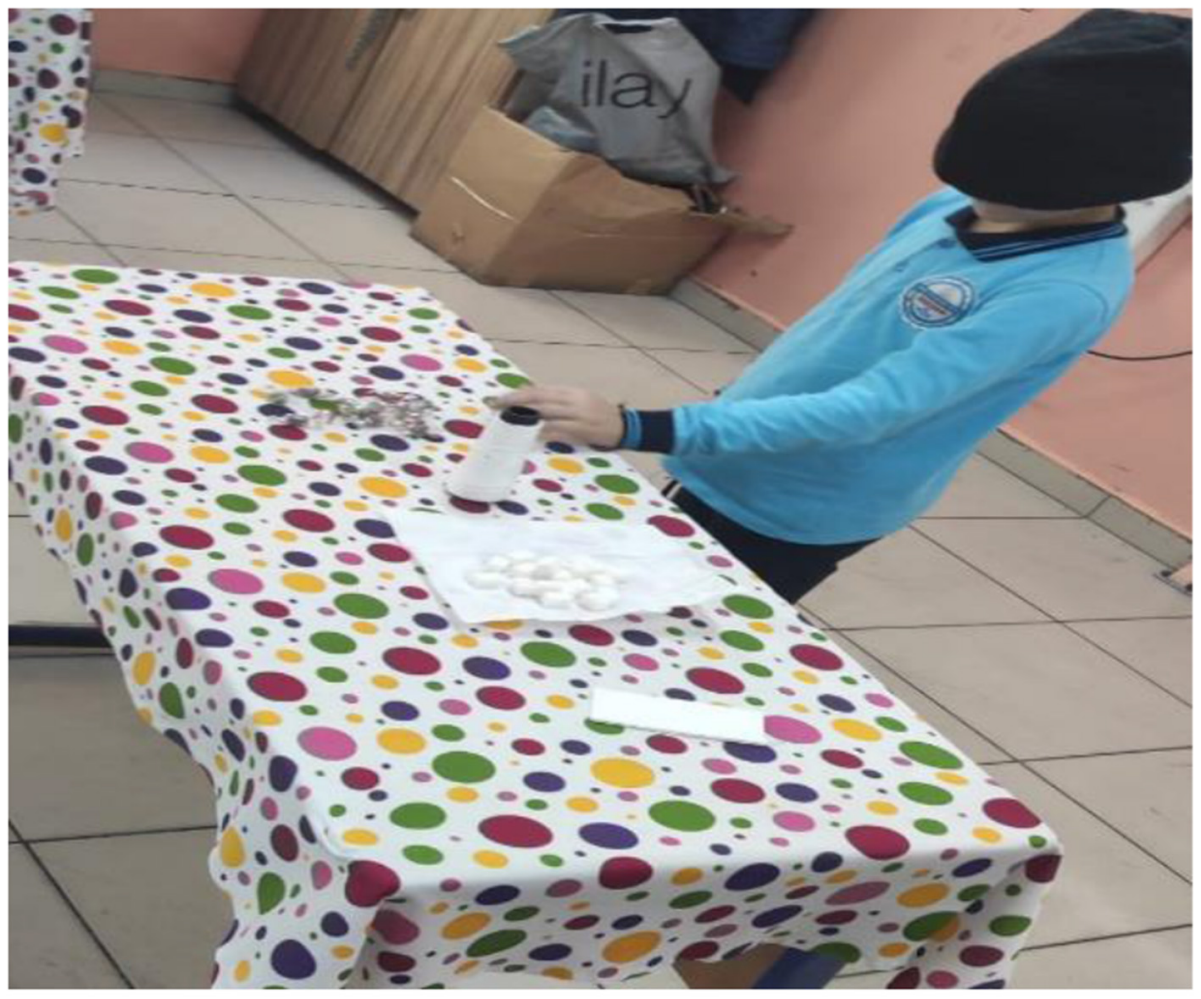
Students examining objects with eyes covered during sensory simulation.

**Figure 4:**
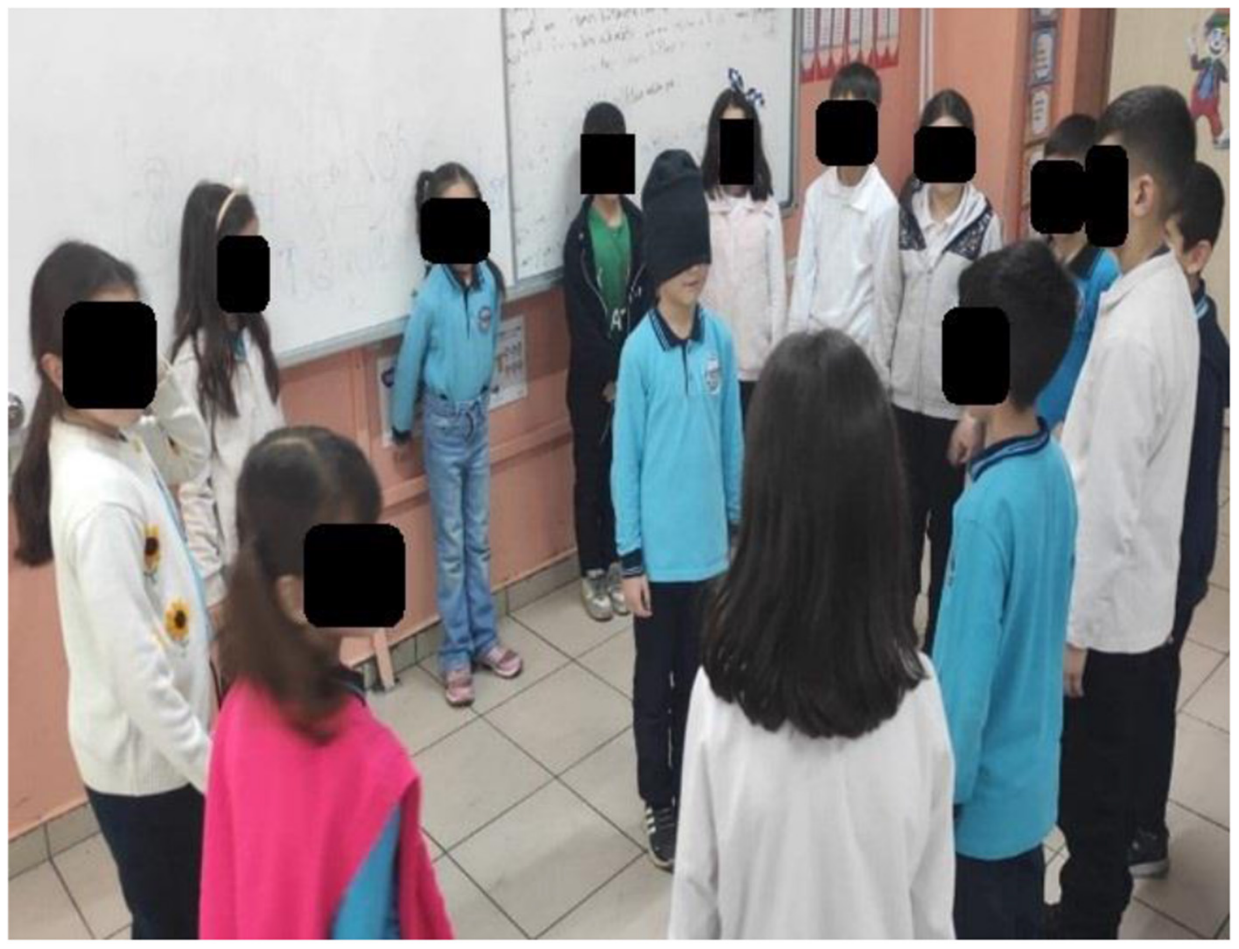
Students identifying classmates' voices while blindfolded.

The preparatory phase involved embodied warm-up activities designed to activate sensory, emotional, or cognitive processes aligned with the weekly theme. For example, during the visual impairment session, students explored classroom objects with their eyes covered in order to foreground tactile perception (see Figure 3). In a related activity, blindfolded students attempted to identify peers solely through auditory cues, shifting attention from visual to auditory processing (see Figure 4). These sensory simulations functioned as experiential entry points into the narrative world of the selected text.

**Figure 5:**
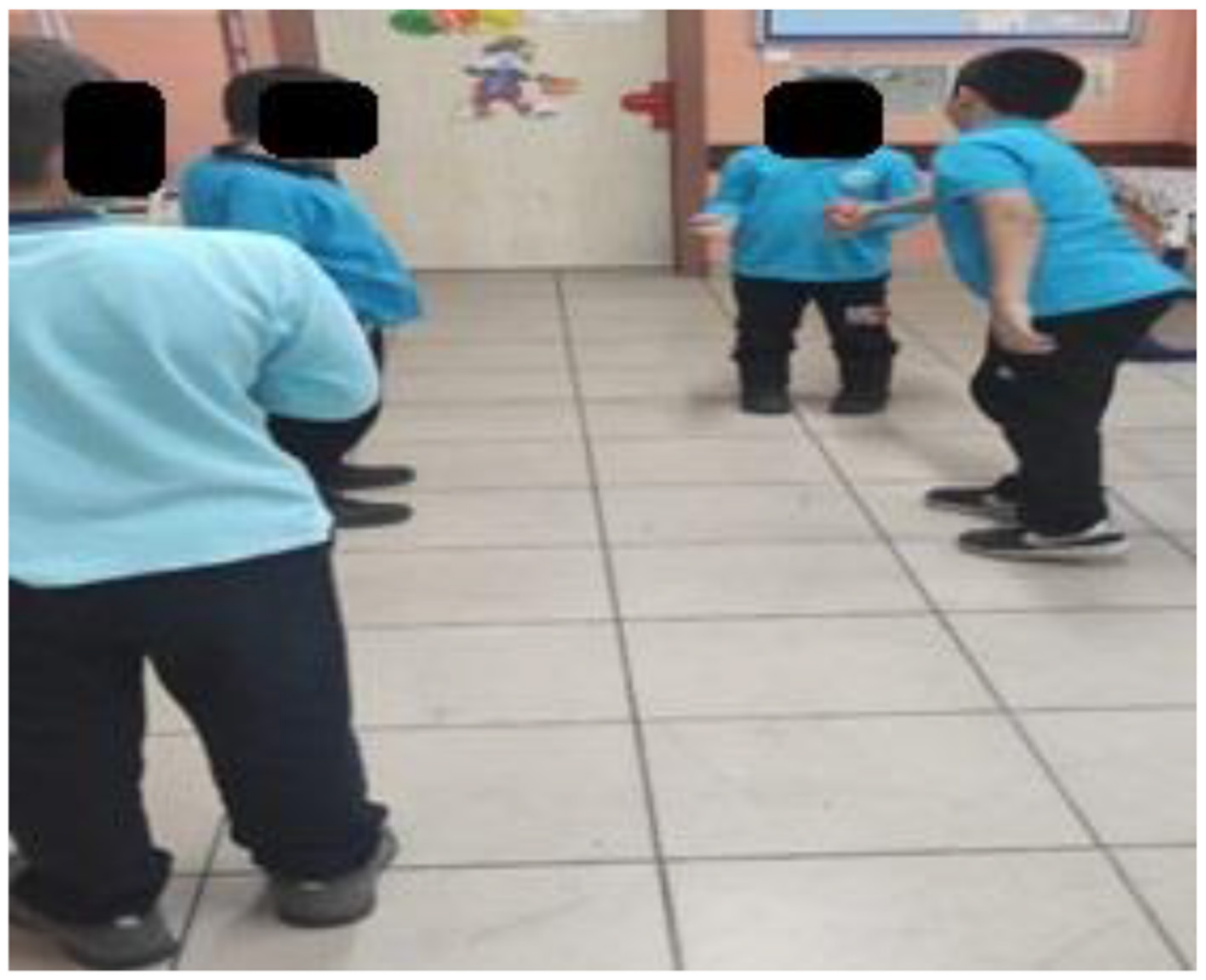
Dramatization of a visually impaired child searching for a lost ball.

**Figure 6:**
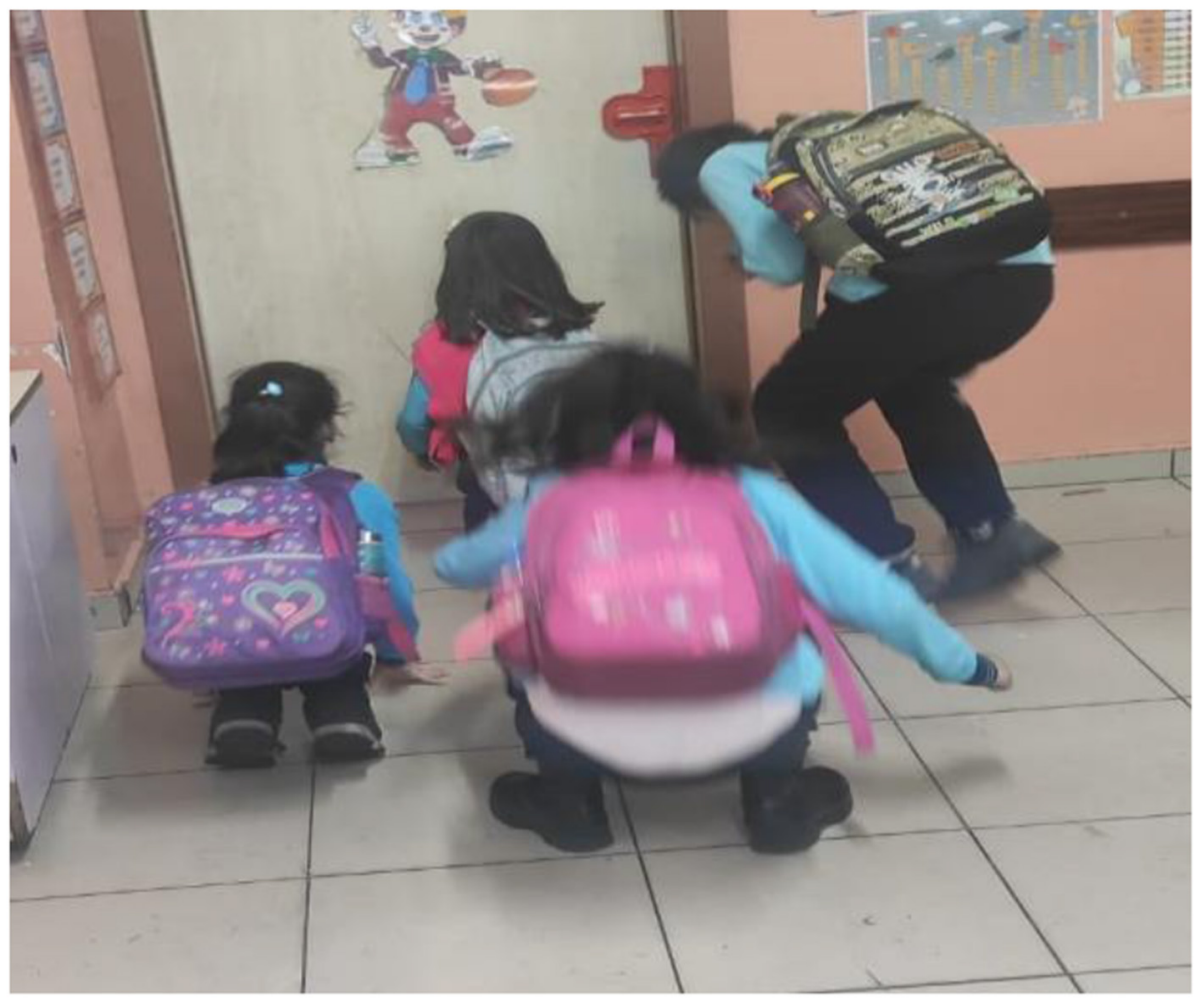
Students enacting migration through symbolic packing and spatial relocation.

Following this preparatory work, the selected book was read aloud interactively. The narrative served as a catalyst for emotional inference, situational analysis, and perspective-taking. Rather than positioning the text as a passive reading task, it was used as the foundation for structured dramatic exploration. The enactment phase constituted the core of each session. A range of creative drama techniques—including role-play, improvisation, frozen image (tableau), teacher-in-role, thought-tracking, inner voice, symbolic representation, and spatial restructuring—were systematically employed to transform narrative comprehension into embodied experience. In the visual impairment session, students dramatized scenarios such as a visually impaired child searching for a lost ball, highlighting spatial uncertainty and reliance on non-visual cues (see Figure 5). Through such scenes, abstract understanding was translated into physical and emotional simulation. Similarly, in the migration-themed session, students embodied displacement through structured improvisation. Representing migrating rabbits, they packed symbolic belongings and physically reorganized classroom space to simulate relocation and spatial restriction (see Figure 6). This spatial reconfiguration allowed students to experience the material and emotional dimensions of forced movement.

The intervention also incorporated a predefined contingency protocol to ensure instructional continuity and emotional safety in cases where the lesson did not proceed as planned. This protocol was shared in advance with the primary school teacher and included structured response strategies for common classroom disruptions such as off-task behavior, emotional over-arousal, communication difficulties, attention fluctuations, unintended stereotyping, and peer conflict. In such cases, the facilitator employed drama-based adjustment strategies rather than removing students from the learning process. These included shifting roles within the drama context, using teacher-in-role techniques to re-engage students, introducing movement-based or observer roles when necessary, pausing and restructuring scenes into tableau form, and facilitating brief reflective discussions to reframe inappropriate representations. Peer mediation and simplified symbolic participation were also used when required.

The evaluation phase constituted a structured and integral component of each weekly session. Rather than functioning as a brief debriefing, this stage was systematically designed to consolidate empathic insight, facilitate emotional regulation, and support cognitive integration of the dramatic experience. Evaluation activities were aligned with the weekly theme and typically included guided whole-class discussion, emotion labeling, perspective-shifting prompts, sentence completion tasks, and collaborative meaning-making exercises. For instance, following the sensory simulation activities in the visual impairment session, students were asked to reconstruct their experience by describing how they navigated uncertainty without visual input. In the dramatization of the lost ball scenario, the class collectively analyzed spatial strategies and emotional responses associated with reliance on non-visual cues. Similarly, after the dyslexia-focused enactments, students engaged in structured discussion about cognitive effort, frustration tolerance, and peer support strategies. In the ADHD session, reflection centered on impulse control, attentional fluctuation, and classroom interaction norms. During the migration-themed week, the symbolic packing activity was followed by dialogue exploring belonging, loss, adaptation, and social inclusion.

Reflection tasks were designed to move from descriptive recounting (“What happened?”) to interpretive analysis (“Why might the character feel this way?”) and finally to application (“How should we respond in real life?”). This progression aimed to activate cognitive empathy, emotional resonance, and prosocial orientation in a sequenced manner. The evaluation phase also ensured safe role exit after emotionally intense dramatizations, thereby supporting students' psychological comfort within the classroom environment.

## 4 Results

The descriptive statistics for pretest and posttest scores across experimental and control groups, including cognitive, emotional, and behavioral empathic tendency subdimensions, are presented in Table 2.

**Table 2:** Pretest and posttest empathic tendency scores by group.

Group	Measure	*n*	Empathic tendency		Cognitive empathy tendency		Emotional empathy tendency		Behavioral empathy tendency	
			*M*	*SD*	*M*	*SD*	*M*	*SD*	*M*	*SD*
Experimental	Pre-test	28	3.05	0.54	3.17	0.71	3.06	0.63	2.92	0.64
	Post-test	28	3.28	0.52	3.33	0.60	3.29	0.59	3.22	0.64
Control	Pre-test	29	2.89	0.54	3.06	0.69	2.77	0.62	2.89	0.62
	Post-test	29	2.76	0.67	2.90	0.79	2.68	0.78	2.72	0.71

As shown in Table 2, students in the experimental group demonstrated higher posttest mean scores than those in the control group across all subdimensions of empathic tendency. This pattern suggests that the drama-based inclusive children's literature intervention was effective in enhancing cognitive, emotional, and behavioral empathic tendencies, providing a clear foundation for the subsequent ANCOVA and MANCOVA analyses.

An ANCOVA was conducted to examine the effect of the drama-based inclusive children's literature intervention on students' total empathic tendency scores while controlling for pretest total empathic tendency. The results are presented in Table 3.

**Table 3:** ANCOVA results for posttest total empathic tendency.

Source	*SS*	df	*MS*	*F*	*p*	Partial η^2^
Pretest total empathic tendency	10.64	1	10.64	61.90	< 0.001	0.53
Group (experimental vs. control)	2.11	1	2.11	12.29	0.001	0.19
Error	9.28	54	0.17			

As shown in Table 3, there was a statistically significant effect of group on posttest total empathic tendency, *F*(1, 54) = 12.29, *p* = 0.001, partial η^2^ = 0.19. After controlling for baseline levels, students in the experimental group demonstrated significantly higher overall empathic tendency scores than those in the control group. The magnitude of the effect was considerable. Pretest total empathic tendency was also a significant covariate, *F*(1, 54) = 61.90, *p* < 0.001, partial η^2^ = 0.53.

To determine whether this overall group difference was reflected across specific dimensions of empathic tendency, MANCOVA was conducted using posttest cognitive, emotional, and behavioral empathic tendency scores as dependent variables, group (experimental vs. control) as the independent variable, and corresponding pretest scores as covariates. The analysis revealed a statistically significant multivariate effect of group on the combined dependent variables, Pillai's Trace = 0.205, *F*(3, 50) = 4.29, *p* = 0.009, partial η^2^ = 0.21. This finding indicates that the inclusive drama-based intervention had a significant overall impact on students' empathic tendencies after controlling for baseline differences. The magnitude of the effect was considerable.

Regarding the covariates, pre-behavioral empathy tendency, Pillai's Trace = 0.252, *F*(3, 50) = 5.63, *p* = 0.002, partial η^2^ = 0.25, and pre-cognitive empathy tendency, Pillai's Trace = 0.306, *F*(3, 50) = 7.36, *p* < 0.001, partial η^2^ = 0.31, significantly predicted posttest empathy tendency scores. However, the multivariate effect of pre-emotional empathy tendency was not statistically significant, Pillai's Trace = 0.141, *F*(3, 50) = 2.73, *p* = 0.053, partial η^2^ = 0.14.

**Figure 7:**
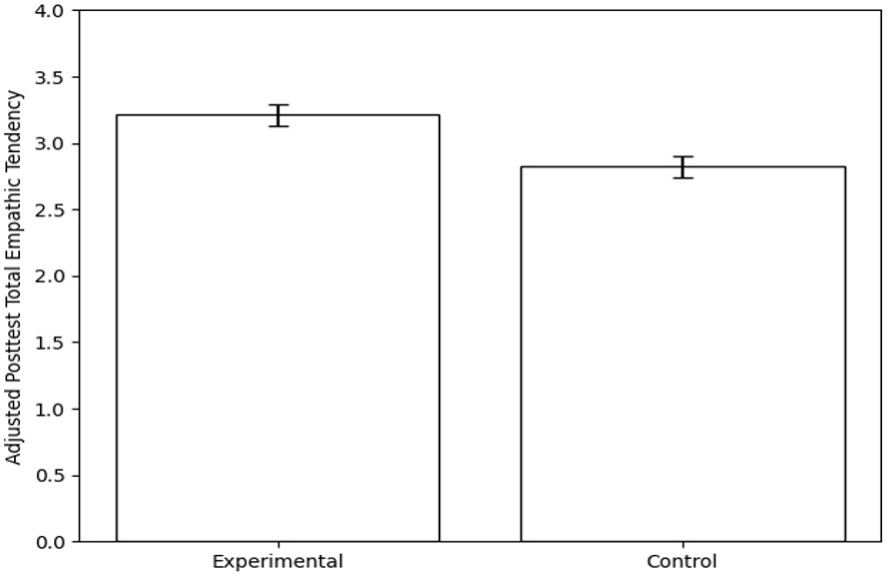
*Adjusted posttest total empathic tendency by group*. (**Note**. Error bars represent standard errors. Adjusted means were estimated controlling for baseline cognitive, emotional, and behavioral empathy scores).

To further illustrate the magnitude and direction of the group difference across the combined empathic tendency dimensions after controlling for baseline scores, adjusted posttest means are presented in Figure 7. As can be seen, the experimental group exhibited higher adjusted posttest total empathic tendency scores than the control group.

Given the significant multivariate effect, follow-up univariate ANCOVA analyses were conducted to determine in which empathy tendency dimensions the intervention produced significant differences. Descriptive statistics and univariate results are presented in Table 4.

**Table 4:** Descriptive statistics and ANCOVA results by group.

Subdimension	Group	*M*	*SD*	*n*	*F*	*p*	Partial η^2^
Cognitive empathy tendency	Experimental	3.33	0.60	28	4.671	0.035	0.082
	Control	2.90	0.79	29			
Emotional empathy tendency	Experimental	3.29	0.59	28	9.871	0.003	0.160
	Control	2.68	0.78	29			
Behavioral empathy tendency	Experimental	3.22	0.64	28	9.922	0.003	0.160
	Control	2.72	0.71	29			

When Table 4 is examined, the experimental group demonstrated higher posttest mean scores than the control group across all empathy subdimensions. For cognitive empathy, the experimental group (*M* = 3.33, SD = 0.60) scored significantly higher than the control group (*M* = 2.90, SD = 0.79), *F*(1, 52) = 4.67, *p* = 0.035, partial η^2^ = 0.08. This finding indicates a moderate effect of the intervention on students' cognitive empathy. For emotional empathy, the difference between the experimental group (*M* = 3.29, SD = 0.59) and the control group (*M* = 2.68, SD = 0.78) was statistically significant, *F*(1, 52) = 9.87, *p* = 0.003, partial η^2^ = 0.16. The effect size suggests a large impact of the intervention on emotional empathy. Similarly, for behavioral empathy, the experimental group (*M* = 3.22, SD = 0.64) scored significantly higher than the control group (*M* = 2.72, SD = 0.71), *F*(1, 52) = 9.92, *p* = 0.003, partial η^2^ = 0.16, indicating a large effect size. Overall, these findings demonstrate that the drama-based inclusive children's literature intervention significantly enhanced students' cognitive, emotional, and behavioral empathy compared to the control condition.

**Figure 8:**
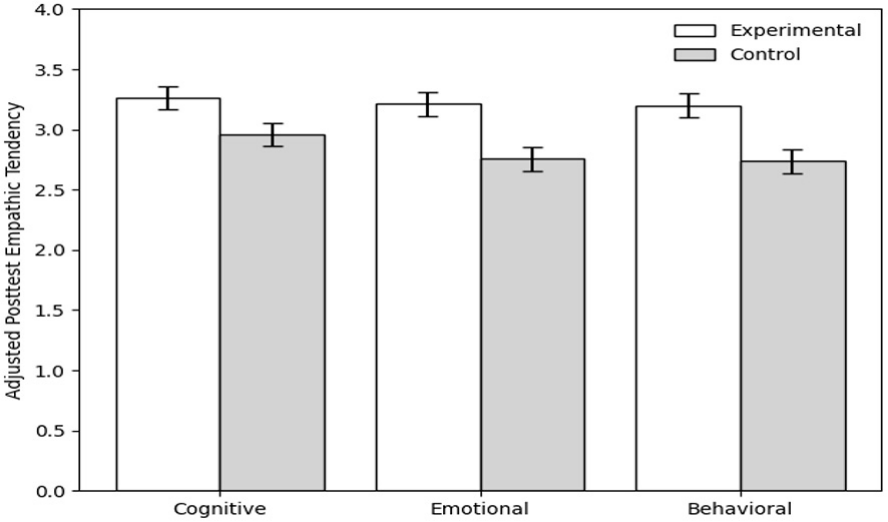
*Adjusted posttest empathic tendency scores by group across subdimensions*. (**Note**. Error bars represent standard errors. Adjusted means were estimated controlling for pretest cognitive, emotional, and behavioral empathic tendency scores).

To provide a clearer visual representation of the adjusted group differences across subdimensions after controlling for pretest scores, the estimated marginal means are presented in Figure 8. As illustrated, the experimental group consistently outperformed the control group across cognitive, emotional, and behavioral empathic tendency dimensions.

## 5 Discussion and conclusion

This study discusses the findings of an original intervention program that examines the effect of the integrated use of inclusive children's literature–based creative drama on the empathic tendencies of fourth-grade primary school students. The results indicate that the developed program significantly increased the total empathic tendency scores of students in the experimental group compared to those in the control group, and that this increase was associated with a large effect size. This finding empirically supports the central hypothesis of the study, which posits that cognitive processes initiated through fictional narratives are transformed into emotional and behavioral outcomes through drama-based experiences. In the literature, empathy is conceptualized as the dynamic interaction of cognitive, emotional, and behavioral components (Cuff et al., 2016; Davis, 1983; Wispé, 1986), and the fact that the intervention yielded significant effects across all sub-dimensions demonstrates the effectiveness of the proposed holistic model.

The Turkish sociocultural context provides a distinctive frame for interpreting the findings of the present study. Turkey's social structure is characterized by a predominantly collectivist cultural orientation, in which values of solidarity, communal responsibility, and relational interdependence are deeply embedded in everyday social life (Hofstede, 2001; Kagitçibaşi, 2007). This collectivist ethos—historically expressed in practices such as the traditional imece tradition of communal labor and reciprocal aid—may have constituted a culturally congruent foundation for the empathy-fostering processes embedded in the intervention. Within such a cultural framework, prosocial dispositions oriented toward collective well-being are not merely introduced through formal pedagogy but are continuously reinforced through family socialization and community participation. In this respect, the drama-based inclusive intervention may have drawn upon and amplified preexisting cultural scripts for solidarity and care, potentially contributing to the comparatively strong effect sizes observed across the emotional and behavioral empathy dimensions. Notably, the Turkish primary school curriculum (Ministry of National Education, 2024). and the social values it promotes—including “love,” “respect,” and “cooperation”—are broadly consonant with the prosocial goals of the intervention, which may have facilitated the internalization of empathic dispositions among participants. Future research should explicitly examine the role of cultural orientation as a moderating variable in empathy-focused educational interventions, in order to determine the extent to which the observed effects are culturally specific or cross-culturally generalizable.

The primary finding of the study is that the integrated use of inclusive children's literature and creative drama significantly enhances students' overall levels of empathy. This finding is consistent with previous research demonstrating the positive effects of drama-based practices on empathy. For instance, Corbett (2019) reported that a drama-based empathy program led to a significant increase in participants' empathy levels. Similarly, Walker and Creely (2025) emphasized that drama-based pedagogies support the development of emotional intelligence and empathy, and identified forum theater as an effective tool for fostering these skills among teachers. These findings highlight the importance of incorporating drama as a pedagogical approach in empathy-oriented classroom practices. In this context, the role of children's literature in fostering empathy is also of critical importance. Children's literature is widely recognized as a powerful tool for supporting children's emotional and social development (Betawi, 2022; Biskin and Hoskisson, 1974; Liang et al., 2025; Lee et al., 2014; Olmstead et al., 2023). For example, in a study grounded in Vygotsky's social constructivist theory and the principles of Universal Design for Learning, Sincuba and Mqondisi (2025) demonstrated that the combined use of drama and inclusive literature facilitated university students' understanding of diversity, strengthened peer relationships, and promoted a sense of belonging, inclusion, and empathic skills.

The observed increase in the cognitive empathy dimension reflects the effect of the intervention program on children's ability to understand characters' intentions, emotions, and thoughts. The moderate effect size identified in the study is consistent with the literature suggesting that children's literature provides rich cognitive schemas that enable children to make sense of diverse life experiences. (Kümmerling-Meibauer 2014) emphasizes that children's literature supports the development of Theory of Mind—a specific cognitive capacity that enables readers to understand others' mental states, including their emotions, thoughts, and intentions. Similarly, Colantonio-Yurko et al. (2021) argue that inclusive picture books can challenge stereotypes, promote the understanding of multiple perspectives, and support more inclusive and equitable classroom environments. Such texts allow students to encounter characters with diverse life experiences, thereby enabling them to view the world from different perspectives (Olmstead et al., 2023). Nikolajeva (2016) further suggests that fictional narratives provide children with cognitive frameworks related to diverse life experiences and facilitate perspective-taking by stimulating imagination. In line with these findings, the meta-analysis conducted by Ciesielska et al. (2025) demonstrated that story-based interventions generally produce a moderate effect on children's empathic skills, thereby supporting the results of the present study. However, the relatively weaker effect observed in cognitive empathy compared to emotional and behavioral dimensions may be attributed to the fact that cognitive empathy primarily relies on rational and mental processes (Davis, 1983). This suggests that cognitive empathy constitutes the starting point of the empathic process, whereas the development of emotional and behavioral dimensions requires experiential learning opportunities.

One of the most striking findings of the study is the strong effect of the intervention on emotional and behavioral empathy. The significant difference observed in the emotional empathy dimension indicates that the intervention effectively supported students' ability to share others' feelings and establish emotional resonance. Emotional empathy involves not only understanding a situation but also “feeling with” others. Kumschick et al. (2014) report that literature-based interventions enhance emotional competence, suggesting that children's literature is a suitable tool for supporting emotional development during middle childhood. Behavioral empathy, on the other hand, refers to the transformation of cognitive understanding and emotional sharing into concrete helping and supportive actions. Bhavnagri and Samuels (1996) highlight that children's literature, along with related drama activities, supports the development of alternative perspectives, thereby influencing social cognition. The role-taking process in drama further concretized these cognitive inferences and enabled students to analyze characters' intentions and beliefs more deeply. This finding is consistent with Corbett's (2019) results, which indicate that drama-based programs not only enhance empathy but also reduce negative behaviors such as bullying, aggression, and victimization. The impact of drama can also be explained through a neurobiological perspective; Prentki (2023) and Keysers (2011) emphasize that the mirror neuron system can be strengthened through such experiential processes and that drama provides a “laboratory” for developing critical empathy. Furthermore, findings by Sun (2025), which demonstrate that drama reduces stigma and promotes inclusion, and by Sincuba and Mqondisi (2025), which show that drama strengthens peer relationships, support the increase observed in behavioral outcomes (e.g., sharing and helping) in this study. As emphasized in Heathcote's approach, when a child “steps into someone else's shoes,” emotional empathy transforms from a temporary feeling into a more enduring sensitivity (Heston, 2013; Mardas and Magos, 2020). It is also likely that the reflection stage included in the intervention allowed students to move beyond the dramatic experience and consider how to transfer these experiences into real-life contexts. Corbett (2019) refers to this process as “open discussion and reflection” and argues that it is central to empathy development. Consistent with the present study, Corbett (2019) also reports that open discussions on empathy support participants in applying this skill to their own lives and in deepening their conceptual understanding. Similarly, Kucirkova (2019) and Kumschick et al. (2014) emphasize that identification with characters yields the most effective outcomes when combined with guided discussions. In the present study, it is therefore plausible that the enactments centered on inclusive themes, together with the subsequent structured discussions, facilitated the transformation of empathic understanding into prosocial behavioral tendencies.

The findings of this study diverge from those reported in some previous studies (Alaca et al., 2023; Çinar et al., 2019). In these studies, drama-based interventions did not produce a significant effect on the empathic tendencies of university-level physiotherapy students. This discrepancy may be explained by differences in developmental stages and intervention design. Fourth-grade primary school students are in the transitional phase from Piaget's concrete operational stage to the formal operational stage, during which perspective-taking abilities undergo significant development. This developmental sensitivity may have created a more favorable context for the effectiveness of empathy-focused interventions. In addition, the integration of the cognitive depth provided by inclusive literary texts with the emotional engagement fostered through drama may have constituted a methodological advantage, leading to stronger effects compared to single-component interventions.

The use of inclusive literature further enhances the value of this study in terms of social justice and social cohesion. The inclusive themes embedded in the intervention program—such as visual impairment, dyslexia, ADHD, physical disability, intellectual disability, and migration—may have played a strategic role in challenging what Hoffman (2000) defines as “similarity bias,” namely the tendency to empathize primarily with individuals who are similar to oneself. Kurtts and Gavigan (2008) argue that children's literature offers deep insights into the lives of individuals with disabilities and that engaging with such narratives fosters sensitivity among children. In the present study, sensory simulations conducted during the visual impairment sessions and the experiential enactments of forced displacement through the “rabbit” metaphor in migration-themed sessions transformed abstract concepts into embodied experiences. As emphasized by Alonge (2024) and Murphy (2024), high-quality representations of disadvantaged groups in literary works play a critical role in shaping children's attitudes toward diversity and enhancing their awareness of “the other.” In particular, dramatized discussions focusing on “invisible disabilities” such as ADHD and dyslexia enabled students to understand the cognitive effort and frustration experienced by their peers, thereby promoting supportive attitudes at the behavioral level. Overall, these themes align with Bishop's (1990) “mirrors, windows, and sliding glass doors” metaphor, offering children opportunities both to recognize their own positionalities and to develop empathic access to the experiences of marginalized groups.

The migration-themed component of the intervention warrants particular contextual attention within the Turkish setting. Turkey currently hosts one of the largest displaced populations in the world, with approximately 3.2 million registered Syrian refugees alongside significant numbers of Afghan and other forced migrants (UNHCR, 2024). This demographic reality is increasingly reflected in the composition of Turkish primary school classrooms; the present study's sample itself included students with Syrian and Afghan migration backgrounds in both the experimental and control groups. The presence of migrant peers in the mainstream classroom constitutes a form of direct intercultural contact that, according to Allport (1954) intergroup contact theory, can reduce prejudice and foster positive intergroup attitudes when appropriate conditions are in place—including equality of status, cooperative goals, and institutional support. Within this context, the migration-themed sessions—in which students enacted forced displacement through the symbolic “rabbit” metaphor and engaged in structured reflection on belonging, loss, and adaptation—were not merely abstract pedagogical exercises but brought participants into experiential contact with socially proximate realities. For students who had directly or indirectly encountered displacement, these dramatized encounters may have carried heightened personal resonance, potentially amplifying the emotional and behavioral empathy outcomes observed in the data. The study thus highlights the particular relevance of culturally responsive, migration-sensitive pedagogy within the Turkish educational context, where the large-scale integration of displaced students into mainstream schooling continues to represent a significant policy and pedagogical challenge.

The originality of this study lies in its integration of inclusive education, children's literature, and creative drama within a unified model. As noted by Metinnam (2025), drawing on Dorothy Heathcote's approach, techniques such as “teacher-in-role” and structured drama sessions provide students with a safe “rehearsal space” in which they can explore social identities and ethical choices. Such a supportive learning environment, as emphasized by Arda-Tuncdemir (2025), encourages students to take risks and engage in new empathic experiences. Accordingly, the findings of this study demonstrate that inclusive literature functions as a cognitive stimulus, while creative drama serves as a pedagogical lever that transforms this cognitive process into emotional and behavioral outcomes.

From a policy perspective, the findings of the present study are particularly meaningful within the evolving landscape of Turkish inclusive education. Turkey's legislative framework for inclusion—most notably the Special Education Services Regulation (Ministry of National Education, 2018)—has progressively expanded the scope of mainstreaming and integration practices by mandating the placement of students with special educational needs in general education classrooms. While this legislative momentum reflects a genuine institutional commitment to inclusive principles, research consistently indicates that implementation remains uneven and that classroom teachers frequently receive insufficient professional preparation to manage the pedagogical demands of inclusive settings effectively (Rakap and Kaczmarek, 2010; Batu et al., 2017). Against this backdrop, the present study demonstrates that a theoretically grounded, literature-based drama intervention—embedded within the existing national curriculum framework through the officially designated Free Activities course—can serve as a practically feasible and institutionally accessible pedagogical response to the implementation challenges of inclusive education in Turkey. The use of literary materials sourced from TÜBITAK and the MoNE further ensures curricular alignment and may facilitate replication by Turkish classroom teachers operating under standard resource constraints. In a system where empathy education is rarely addressed through explicit and structured programming, the proposed integrated model offers a replicable and evidence-based framework that is consonant with both national educational priorities and international inclusive education commitments to which Turkey is a signatory (UNESCO, 1994).

In conclusion, creative drama practices integrated with inclusive children's literature foster the multidimensional development of empathy among primary school students. Awareness initiated at the cognitive level gains emotional depth through drama and ultimately translates into behavioral tendencies. This integrated model can be recommended as an effective pedagogical strategy for promoting inclusive school cultures and supporting students' social-emotional development.

### 5.1 Implications for practice

The findings of this study offer several important implications for educational practice, particularly in the design of inclusive and empathy-oriented learning environments. First, the results highlight the pedagogical value of integrating inclusive children's literature with creative drama in classroom settings. Teachers can use carefully selected inclusive texts as a starting point to introduce diverse perspectives and initiate cognitive empathy, and then extend this process through drama-based activities that enable students to embody and experience these perspectives. This integrated approach can support the development of empathy not only at a cognitive level but also at emotional and behavioural levels. In addition, the study underscores the importance of using structured and process-oriented drama practices rather than isolated or unstructured activities. Incorporating stages such as warm-up, enactment, and reflective discussion allows students to internalize their experiences and connect them to real-life situations. Therefore, teachers should be supported in designing and facilitating structured drama sessions that include opportunities for reflection and dialogue. Another important implication is that, the use of inclusive themes (e.g., disability, migration, learning differences) in instructional materials can contribute to fostering respect for diversity and reducing bias among students. Teachers are encouraged to intentionally select and integrate such themes into their curricula to promote inclusive attitudes and social awareness from early grades.

### 5.2 Limitations and recommendations for future research

Despite its contributions, this study has several limitations. First, the research was conducted within a single school context with a relatively limited sample of fourth-grade students (*N* = 57), which restricts the generalizability of the findings. Future studies should replicate the intervention across different regions, school types, and cultural contexts to examine its broader applicability. Second, the study relied primarily on self-report measures to assess empathic tendencies. Although validated instruments were used, self-reports may be influenced by social desirability and may not fully reflect actual behavioral outcomes. Future research should incorporate multiple data sources, such as observations, peer reports, and qualitative data, to provide a more comprehensive understanding of empathy development. Third, the intervention was limited in duration and did not include a follow-up measurement. Therefore, the long-term sustainability of the observed effects remains unclear. Longitudinal studies are needed to examine whether the gains in cognitive, emotional, and behavioral empathy persist over time.

## Data Availability

The raw data supporting the conclusions of this article will be made available by the authors, without undue reservation.

## Author's note

This study was presented as an oral presentation at the 24th International Primary Teacher Education Symposium held on 14–17 May 2026 in Istanbul, Türkiye.
